# OWL 2 learn profile: an ontology sublanguage for the learning domain

**DOI:** 10.1186/s40064-016-1826-0

**Published:** 2016-03-08

**Authors:** Sudath R. Heiyanthuduwage, Rolf Schwitter, Mehmet A. Orgun

**Affiliations:** Department of Computing, Faculty of Science and Engineering, Macquarie University, Sydney, NSW 2109 Australia

**Keywords:** Ontology, Semantic web, Knowledge representation, Learning information systems

## Abstract

Many experimental ontologies have been developed for the learning domain for use at different institutions. These ontologies include different OWL/OWL 2 (Web Ontology Language) constructors. However, it is not clear which OWL 2 constructors are the most appropriate ones for designing ontologies for the learning domain. It is possible that the constructors used in these learning domain ontologies match one of the three standard OWL 2 profiles (sublanguages). To investigate whether this is the case, we have analysed a corpus of 14 ontologies designed for the learning domain. We have also compared the constructors used in these ontologies with those of the OWL 2 RL profile, one of the OWL 2 standard profiles. The results of our analysis suggest that the OWL 2 constructors used in these ontologies do not exactly match the standard OWL 2 RL profile, but form a subset of that profile which we call OWL 2 Learn.

## Background

An ontology is a conceptual specification of a domain that represents concepts, relations and constraints of that domain. A well designed learning ontology helps to clearly represent a learning domain (LD) and eventually could be used to search for learning resources. Moreover, the use of a learning ontology in an online learning system could assist students who ask questions and search for learning resources by using the domain knowledge specified in the ontology. Different learning ontologies have already been designed for various purposes for some higher educational institutions.

Early attempts on learning ontologies focused more on conceptual modelling of learning ontologies. For example, a topic map ontology for e-learning has been proposed by Kolås (2006) to share learning resources. UML (unified modelling language) diagrams have been used by Knight et al. (2006) who propose an ontology-based approach for adaptive and flexible learning. Some of the learning ontologies that have been proposed later have focused more on ontology design, using design tools and the web ontology language (OWL) (Hitzler et al. 2012). OWL and OWL 2 have been used to specify various aspects of learning ontologies. For example, a learning ontology has been used to measure the semantic relevance between a learning resource and the learning context of a learner (Yessad et al. 2011). In this work, concept maps have been used initially to model an ontology and then OWL to build the ontology for measuring the semantic relatedness for relevance ranking of learning resources. OWL/OWL 2 based ontologies have also been used to recommend the contents in a tutoring system (Vesin et al. 2013). The ontologies of this work (ontologies for learning resources, tasks, learner model and teaching strategy) have been developed using the ontology design tool Protégé. An OWL-based ontology for teaching mathematical word problems has been proposed by Lalingkar et al. (2014). These studies show the increasing popularity of OWL/OWL 2 for implementing learning ontologies.

In a recent study, the Higher Education Reference Ontology (HERO) developed in OWL (*HERO_ONTOLOGY_V 25.06.2013.owl*) has been proposed to overcome the problems in building application specific ontologies in the higher education domain (Zemmouchi–Ghomari and Ghomari 2012). This study has found that the development and interoperability of application specific ontologies are difficult. Therefore, we believe that the identification of a set of common features (OWL/OWL 2 constructors) in existing ontologies of the LD might help ontology designers in their work. However, we could not find any studies in the literature that attempt to identify a common set of OWL 2 constructors for the LD.

The World Wide Web Consortium (W3C) has recommended OWL 2 as a standard ontology language for the Semantic web which is based on a particular version of description logic (DL) (Hitzler et al. 2012). The W3C has also recommended three standard profiles: OWL 2 EL, OWL 2 QL, and OWL 2 RL that are targeted at different application areas (Motik et al. 2012). Each standard OWL 2 profile includes a subset of OWL 2 constructors and has different computational properties (Motik et al. 2009).

To the best of our knowledge, no one has investigated so far how well learning ontologies are aligned with any of these three standard OWL 2 profiles. This paper aims at answering this question and identifying a common subset of OWL 2 constructors (a sublanguage or a profile) for the LD. In our study, we have first collected and analysed a corpus of 14 learning ontologies that have been developed in OWL/OWL 2, including one in RDF/RDFS. When we consider the ontologies in our corpus, we can find a lot of object and/or data properties that require an expressive version of DL. Hence, one could presume that the OWL 2 RL profile is a good starting point for modelling the LD. However, if it is the case that the learning ontologies in our corpus have different features, then it would make sense to propose a new OWL 2 profile for the LD.

We expect that modelling a LD requires different institution-specific ontologies whose expressive power depends on specific applications. For example, one of the ontologies in our corpus, the university ontology (*university.owl*^1^), is based on a highly expressive DL language. This ontology includes nominals (individual names) and cardinality restrictions (counting quantifiers) which increase the expressivity of the underlying DL language. On the other hand, another ontology in our corpus, the university benchmark ontology [*uni*-*bench.owl* (see Footnote 1)], does not include nominals or cardinality restrictions. That means that the university benchmark ontology is based on a less expressive DL language than the language of the university ontology. This is not surprising, since these two learning ontologies have been designed to satisfy different institutional requirements resulting in different ontology structures and features.

The study reported in this paper substantially extends a preliminary analysis of a corpus of ten learning ontologies (Heiyanthuduwage et al. 2014). In our study, we have first collected a corpus of 14 ontologies designed for the LD, including several developed in OWL 2. We then identify and analyse the usage of OWL/OWL 2 constructors in our corpus and compare these identified constructors with those of the OWL 2 RL profile. We observe that not all the constructors in the OWL 2 RL profile are used in our corpus. Finally, we introduce the resulting new profile called OWL 2 Learn and investigate its expressivity.

The rest of this paper is structured as follows. In “Ontology languages and ontology language profiles” section, we introduce ontology languages and the three standard OWL 2 profiles. In “The corpus of LD ontologies, characteristics and implications” section, we discuss the corpus of 14 learning ontologies and provide an analysis of the corpus. In “Analysis of the corpus and findings” section, we discuss the findings of the analysis of the ontology corpus. In “OWL 2 constructors of the corpus and the OWL 2 RL profile” section, we present a comparison of the constructors found in the corpus and the constructors of the OWL 2 RL profile. In “OWL 2 learn profile” section, we introduce the new OWL 2 Learn profile and discuss its expressive power. In “Conclusion” section, we summarize our contribution and discuss future work directions.

## Ontology languages and ontology language profiles

An ontology can formally be specified using an ontology language. Different ontology languages have been proposed over the last two decades. The Resource Description Framework (RDF) has been accepted by the W3C in 2004 as a framework for describing resources on the Web (Cyganiak et al. 2014). However, RDF does not include sufficient constructors to specify a comprehensive ontology. RDFS, a schema language for RDF, provides a framework for describing application-specific classes and properties (Horrocks 2001). However, OWL superseded RDF/RDFS in 2004 as a Web ontology language and is now a W3C recommendation for the Semantic Web. As OWL is based on a version of description logic, it allows the use of a DL-based reasoner to derive information that is not explicitly specified in an OWL ontology (Horrocks and Patel–Schneider 2011). Since 2009, OWL 2 has been used as the W3C recommended ontology language for the Semantic Web (Motik et al. 2009). OWL 2 is a new and more expressive version of OWL which mainly improves the relational and datatype expressivity of the language.

### OWL, OWL 2 and their expressivity

The expressivity of the underlying DL language is a distinct feature of an ontology language and is determined by the type of constructors that are allowed in the language and how these constructors can be combined. Over the last two decades, the main focus of DL research was to increase the expressive power of DL languages and to understand their formal properties (Baader et al. 2010). Highly expressive ontology languages include many types of different constructors. However, high expressivity comes at a price and query answering over expressive ontologies can be computationally expensive.

The ontology languages RDF/RDFS, OWL and OWL 2 show a gradual increase in expressive power. OWL includes a range of constructors and axioms that provide a higher expressivity than RDF/RDFS.

OWL 2 includes a number of extensions to OWL such as new constructors for expressing additional restrictions and characteristics of properties and property chains and keys (Motik et al. 2012). Self-restriction ObjectHasSelf() is one of them. OWL includes only three constructors for non-qualified cardinality restrictions as shown in (1) below whereas OWL 2 includes constructors for both non-qualified and qualified cardinality restrictions as shown in both (1) and (2) below.

1 $$ \left( {{\texttt{ObjectMaxCardinality}}\left( {{\texttt{n }}\ \texttt{R}} \right),{\texttt{ ObjectMinCardinality}}\left( {\texttt{n\ R}} \right),{\texttt{ ObjectExactCardinality}}\left( {\texttt{n\ R}} \right)} \right) $$

2 $$ \left( {{\texttt{ObjectMaxCardinality}}\left( {\texttt{n\ R\ D}} \right),{\texttt{ ObjectMinCardinality}}\left( {\texttt{n\ R\ D}} \right),{\texttt{ ObjectExactCardinality}}\left( {\texttt{n\ R\ D}} \right)} \right) $$

OWL 2 also includes different constructors for object properties and data properties. For example, OWL includes a single constructor rdfs:domain() to specify both the object property domain and the data property domain whereas OWL 2 includes two separate constructors ObjectPropertyDomain() and DataPropertyDomain() to specify the object property domain and the data property domain respectively.

OWL includes the constructor DisjointClasses(C_1_C_2_) to specify disjoint classes. In addition to the above, OWL 2 introduces two constructors DisjointObjectProperties() and DisjointDataProperties() to specify disjoint object properties and disjoint data properties respectively. OWL 2 also introduces the constructor ObjectPropertyChain() to help specify property chains and the constructor HasKey() to define unique keys. OWL 2 includes extended datatypes; for example, owl:real and owl:rational. OWL 2 also provides additional features on data types that include datatype restrictions, range of datatypes, datatype definitions, new datatypes, and data range combinations. Data ranges can be combined by means of intersection, union and complement. Another new feature of OWL 2 is punning, that is, using the same name for a class and an individual or for properties and individuals or classes and object properties.

Even though OWL includes property assertions, it does not distinguish between object and data property assertions. For example, OWL uses the constructor samePropertyAs(PN a_1_a_2_/v) for both object and data property assertions. On the other hand, OWL 2 includes two separate constructors ObjectPropertyAssertion(PN a_1_a_2_) and DataPropertyAssertion(R a v) for object property assertion and data property assertion, respectively.

It has been shown that OWL has the expressivity of the DL language *SHOIN(D)* (Horrocks et al. 2003). OWL 2 is more expressive than OWL as it supports complex property inclusion axioms. It also includes new constructors to gain syntactic freedom; for example, it allows ontology designers to use DisjointUnion and DisjointClasses to express disjointness in a more compact way (Golbreich et al. 2012). Overall, OWL 2 has the expressivity of the DL language *SROIQ(D)* which is strictly more expressive than the DL language *SHOIN(D)* (Horrocks et al. 2006).

### OWL 2 standard profiles and learning ontologies

In recent years, research on DL based ontology languages has paid an increasing attention to identifying sublanguages to specify different types of application domains that require restricted expressivity. OWL profiles include subsets of OWL 2 constructors and are designed for particular applications and reasoning tasks. The OWL 2 EL profile is suitable for ontologies with a large number of concepts and/or properties for which the basic reasoning tasks require polynomial time in terms of the size of the ontology (Motik et al. 2012). The OWL 2 QL profile is recommended for applications that work on large volumes of data where the query answering tasks require logarithmic space in terms of the size of the data (Motik et al. 2012). The OWL 2 RL profile has been proposed for domains that require scalable reasoning but do not require too much expressive power compared to full OWL 2. OWL 2 RL implementations can use rule-based reasoning engines and query answering over OWL 2 RL ontologies require polynomial time in terms of the size of the ontology (Motik et al. 2012).

As we can see, each OWL 2 standard profile has been recommended for specific types of applications. We could not find any works in the literature that discuss the applicability of OWL 2 standard profiles to the learning domain. Therefore, as a starting point, it is worth analysing the OWL 2 constructors used in proposed learning ontologies and investigate the required expressivity of the DL language which can be used to model this domain.

## The corpus of LD ontologies, characteristics and implications

The corpus of the learning ontologies that we collected for our analysis consists of 14 ontologies (Table 1). 12 of these ontologies are publicly available and have been developed by researchers for use at different institutions. In addition to these 12 ontologies, the corpus includes two ontologies that we have developed for Charles Sturt University and Macquarie University. This section describes the characteristics of this corpus.

**Table 1:** The corpus of learning ontologies

#	Ontology file name	Institution
1	university.owl	Manchester University
2	univ-bench.owl	Leehigh University
3	AIISO schema-20080925.owl	Talis Information Ltd
4	swrc_v0.3.owl	University of Karlsruhe
5	TMDU.owl	Tokyo Institute of Technology
6	HU.owl	Tokyo Institute of Technology
7	TITech.owl	Tokyo Institute of Technology
8	ecs.owl	University of Southampton
9	AcademicInstitute.rdfs	University of Aberdeen
10	lom.owl	University of Alcala, Pontifical University of Salamanca
11	HERO_ONTOLOGY_V 25.06.2013.owl	M’hammed Bouguerra Boumerdès University
12	instOntology.owl	Indian Statistical Institute
13	CSU_Ontology.owl	Charles Sturt University
14	MQ_Ontology.owl	Macquarie University

### RDF/RDFS and OWL/OWL 2 ontologies

We identified a number of learning ontologies in OWL/OWL 2 format and in RDF/RDFS format in the open domain. Only one RDF/RDFS ontology (*AcademicInstitute.rdfs*) was included in the corpus. This might be, because RDF/RDFS is not very expressive and has been outdated by OWL/OWL 2 in recent years. We came across many other learning ontologies discussed in research papers; however, they were not included in the corpus, because we do not have access to the full ontologies.

### Syntax of OWL 2 ontologies

The ontologies in our corpus use three different syntaxes: OWL/XML syntax, OWL functional-style syntax and Turtle syntax. Although the choice of the syntax provides some flexibility for the ontology designer, it makes searching for OWL/OWL 2 constructors in the corpus difficult. For our analysis, we searched for OWL/OWL 2 constructors in all these three different syntaxes.

**Fig. 1:**
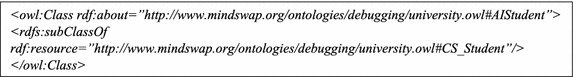
A class defined in OWL/XML syntax

The most commonly used syntax in our corpus is based on OWL/XML. This makes the ontologies machine-readable but also very verbose. For example, the *university.owl* ontology uses the constructor rdf:subClassOf(C_1_, C_2_) to state that an artificial intelligence student (AIStudent) is a computer science student (CS_Student) as in (Fig. 1). The same statement can be expressed more concisely in the DL notation (3) or in the OWL 2 functional-style syntax (4):

3 $${\texttt{AIStudent}} \subseteq {\texttt{CS}}\_{\texttt{Student }}$$

4 $${\texttt{SubClassOf}}\left( {{:}{\texttt{AI}}\_{\texttt{Student }}{\,:}{\texttt{CS}}\_{\texttt{Student}}} \right)$$

**Fig. 2:**
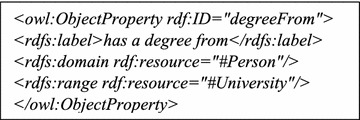
An object property defined in OWL/XML syntax

Similarly, the *uni*-*bench.owl* ontology uses the constructor ObjectProperty(C_1_, C_2_) to state that a person has got a degree from a University as in (Fig. 2). This statement can also be written in functional-style syntax as shown in (5) below:

5 $${\texttt{degreeFrom ({:}Person :University)}}$$

In the following discussion, we present our examples in the DL notation or in the OWL 2 functional-style syntax.

### Use of RDF constructors in OWL/OWL 2 ontologies

All ontologies of the corpus include OWL/OWL 2 constructors. In addition, some ontologies include RDF/RDFS constructors as well. RDF/RDFS constructors are used to specify domain information that refers to RDF resources (Carroll et al. 2012). For example, some named classes are defined with the help of the OWL 2 constructor owl:Class. Some named classes that are RDF resources are defined with the help of the RDF/RDFS constructor rdfs:Class. In addition to rdfs:Class, a number of additional RDF/RDFS constructors have been used in some ontologies; for example, rdf:DataType, rdfs:subClassOf, rdfs:subPropertyOf, etc. The reason for this is that semantic web languages are layered and those at a higher level borrow constructors from those at a lower level to avoid redundancy (W3C 2013; Carroll et al. 2012).

## Analysis of the corpus and findings

This section provides a detailed discussion of our analysis of the corpus. In order to identify the OWL/OWL 2 constructors in the corpus, we used a pattern matching program. In the following, we discuss the usage patterns of OWL/OWL 2 constructors in the corpus and the DL expressivity of the corpus.

### The usage patterns of OWL/OWL 2 constructors in the corpus

Different OWL/OWL 2 constructors have been used within the corpus to express different aspects of the LD. We first identified whether each OWL/OWL 2 constructor was used in the corpus or not. If a constructor was used, then we recorded its location and its frequency.

In this analysis, we used two main metrics to measure the usage of the constructors: a score and a frequency. Score (n) is the number of ontologies within the corpus where a given OWL 2 constructor is used. Frequency (f) is the percentage of the use of a given OWL 2 constructor within the corpus. Frequency is calculated as (the total number (sum) of occurrences of a given constructor within the corpus)/(the total number of occurrences of all the constructors in the corpus)×100. Both metrics are required, because a particular constructor may have been used a lot in a large ontology whereas the same constructor may have not been used much in a small ontology (Power and Third 2010). For example, the constructor disjointClasses(C_1_C_n_) has a frequency of 8.85 % (sum = 544) whereas the constructor dataProperty(P_1_P_n_) has a frequency of 16.79 % (sum = 1032) (Table 2). However, the score for both constructors is nine, since they occur in the same number of ontologies. We have observed that most of the constructors that have higher scores also have higher frequencies.

**Table 2:** Commonly used and infrequently used OWL/OWL 2 constructors in the corpus

**#**	OWL 2 Constructor/s	DL Syntax	DL Example	n	f	sum
Commonly used OWL 2 constructors						
1	Class(C)	A, C, D	CS_Student	14	12.27	754
2	SubClassOf(C1 C2)	C ⊑ D	CS_Student ⊑ Student	14	12.41	763
3	ObjectProperty(P)	P	advisorOf	12	10.85	667
4	DisjointClasses(C1 C2) DisjointClasses(C1 Cn)	C_1_⊔…⊔C_n_	AI_Student ⊔ HCI_Student	9	8.85	544
5	rdfs:DataType(), DataProperty(D), DataTypeProperty()	D	hasTenure Boolean, NonNegativeInteger	9	16.79	1032
6	SubObjectPropertyOf(P1 P2), SubPropertyOf(P1 P2)	H—Role hierarchy	doctoralDegreeFrom ⊑ degreeFrom	8	5.60	344
Infrequently used OWL 2 constructors						
7	SomeValuesFrom(P C), ObjectSomeValuesFrom(P C)	∃P.C	∃hasAdvisor.PhDStudent	7	1.59	98
8	AllValuesFrom(P C), ObjectAllValuesFrom(P C)	∀P.C	∀takesCourse.CS_Course	6	1.40	86
9	owl:Thing	$\top$	Class: Thing	7	1.09	56
10	ObjectIntersectionOf(C1…Cn), IntersectionOf(C1…Cn)	C_1_⊓…⊓C_n_	CS_Department ⊓ hasResearchArea.AI	6	1.27	78
11	MaxCardinality(n R D), MinCardinality(n R D), ExactCardinality(n R D)	(≥ nR) (≤ nR)	≥3 takesCourse.CS_Course ≤ 1 takesCourse.CS_Course	6	1.16	71
12	EquivalentClass(C1…Cn), EquivalentClasses(C1…Cn)	C_1_ ≡ C_2_	AI_Academic ≡ CS_Department ⊓ hasResearchArea.AI	6	0.86	53
13	ObjectUnionOf(C1…Cn), UnionOf(C1…Cn)	C_1_⊔…⊔C_n_	owl_SemanticLink ⊔ oc_SemanticLink	6	0.57	35
14	InverseObjectProperties(P1 P2), InverseOf(PN)	P^−^	advisorOf ^−^ ≡ hasAdvisor	8	0.46	28
15	ObjectHasValue(P a), DataHasValue(R v), hasValue(), ObjectOneOf(a1…an), oneOf	∃R. {x}, {x_1_, …, x_n_}	∃hasResearchArea. {AI}, {A26, A27}	4	0.23	14
16	TransitiveObjectProperty	P transitive role	SubOrganisation of is a transitive role	4	0.18	10

Each OWL/OWL 2 constructor used in an ontology corresponds to a specific DL constructor. The corpus also includes multiple OWL/OWL 2 constructors that correspond to the same DL constructor with a specific DL expressivity. We list OWL/OWL 2 constructors found in the corpus with the corresponding DL syntax (Tables 2, 4). In our work, some counts (n) were taken on individual constructors and some on OWL 2 constructor groups, each of which corresponds to a specific DL constructor. For example, the three OWL 2 constructors for qualified cardinality restriction that are given in “Analysis of the corpus and findings” section correspond to a single DL constructor of qualified cardinality restriction (Q). Again, both RDF/RDFS constructor rdfs:Class and OWL/OWL 2 constructor owl:Class refer to the same DL constructor of Atomic Concept (A).

Based on the score and frequency of OWL 2 constructors or constructor groups, we found that there are three identifiable categories of constructors: (1) *commonly used constructors*, (2) *infrequently used constructors*, and (3) *unused constructors*. In the following subsections, we discuss each of these categories of constructors in more detail.

#### Commonly used OWL/OWL 2 constructors

Our analysis shows that some OWL/OWL 2 constructors have been commonly used in a majority of ontologies. The OWL 2 constructors or constructor groups that have a score of greater than or equal (≥) to 8 and a frequency of greater than (>) 5 % were included in this category (Table 2). The constructors Class() and SubClassOf(C, D) have the highest score of 14 and frequencies of 12.27 and 12.41 %, respectively. The constructor SubPropertyOf(P_1_ P_2_) has the smallest score of eight within this category and a frequency of 5.6 %.

#### Infrequently used OWL/OWL 2 constructors

In our analysis, we have also identified a set of infrequently used OWL/OWL 2 constructors. These OWL/OWL 2 constructors or constructor groups have a score of less than (<) 8 and a frequency of less than (<) 5 % (Table 2). They also have a low sum that is below 100. These measures show that infrequently used OWL/OWL 2 constructors are not required much within the corpus. Furthermore, all these OWL/OWL 2 constructors have a frequency below 2 % and some of them have frequencies even below 1 % (Table 2).

#### Unused OWL/OWL 2 constructors

OWL 2 provides many constructors to specify concepts, object properties, and data type properties. However, we have found that some OWL/OWL 2 constructors are not used within the corpus; for example, object and data complement constructors: ObjectComplementOf() and DataComplementOf(). None of the ontologies in the corpus specified individual values and reflexivity. Furthermore, the ontologies in the corpus did not use some of the data and object properties. More details of the unused OWL/OWL 2 constructors are provided in “OWL 2 RL constructors not used in the corpus” section.

### The expressivity of LD ontologies of the corpus

We can measure the diversity of an ontology based on the number of different OWL/OWL 2 constructors used in a single ontology. A higher diversity means that a wide variety of OWL/OWL 2 constructors is used in that ontology. However, it is possible that some OWL/OWL 2 constructors or constructor groups are slight variations that refer to the same DL constructor. In such a situation, the DL expressivity of the ontology would not change. For example, all the OWL 2 constructors on qualified cardinality restrictions refer to the same DL expressivity (*Q*). Based on the different types of OWL/OWL 2 constructors used in each ontology of the corpus, we identified the DL expressivity of these ontologies (Table 3).

**Table 3:** An analysis of the expressivity of the corpus

#	Ontology file name	Diversity	*FL* ^−^	*R*+	*H*	*I*	*N/Q*	*O*	*D*	Expressivity
1	TMDU.owl	11	*√*						*√*	*FL* ^−^ *(D)*
2	TITech.owl	12	*√*						*√*	*FL* ^−^ *(D)*
3	HU.owl	11	*√*						*√*	*FL* ^−^ *(D)*
4	AcademicInstitute.rdfs	9	*√*		*√*				*√*	*FL* ^−^ *H(D)*
5	swrc_v0.3.owl	31	*√*			*√*			*√*	*FL* ^−^ *I(D)*
6	AIISO schema-20080925.owl	21	*√*		*√*	*√*			*√*	*FL* ^−^ *HI(D)*
7	univ-bench.owl	21	*√*	*√*	*√*	*√*			*√*	*FL* ^−^ *R* ^+^ *HI(D)*
8	MQ_Ontology.owl	27	*√*	*√*	*√*	*√*	*Q*		*√*	*FL* ^−^ *R* ^+^ *HIQ(D)*
9	CSU_Ontology.owl	23	*√*	*√*	*√*	*√*	*Q*		*√*	*FL* ^−^ *R* ^+^ *HIQ(D)*
10	HERO_ONTOLOGY_V 25.06.2013.owl	13	*√*			*√*	*Q*	*√*	*√*	*FL* ^−^ *OIQ(D)*
11	ecs.owl	21	*√*		*√*		*N*	*√*	*√*	*FL* ^−^ *HON(D)*
12	instOntology.owl	22	*√*			*√*	*N*	*√*	*√*	*FL* ^−^ *OIN(D)*
13	lom.owl	32	*√*			*√*	*N*	*√*	*√*	*FL* ^−^ *OIN(D)*
14	university.owl	32	*√*	*√*		*√*	*N*	*√*	*√*	*FL* ^−^ *R* ^+^ *OIN(D)*

The diversity of the ontologies in the corpus varies from 9 to 32 whereas their expressivity varies from *FL*^−^*(D)* to *FL*^−^*R*^+^*ION(D)*. Still, we further analysed the usage of OWL/OWL 2 constructors used in the corpus to reveal a broader view of the OWL/OWL 2 constructors within the corpus. All the learning ontologies of the corpus include OWL/OWL 2 constructors that refer to the Frame Language (*FL*^−^). *FL*^−^ includes intersection of concepts, value restrictions and simple existential quantification (Baader et al. 2003). *FL*^−^ is a sublanguage of the Attributive Language (*AL*) that is obtained by disallowing atomic negation from *AL* (Baader et al. 2003). *AL* includes the features of atomic concept, universal concept, bottom concept, intersection, value restriction, limited existential quantification and atomic negation (Baader and Nutt 2003). If full negation/complement (*C*) is included, then we end up with the DL language *ALC*.

We have found that transitive roles (*R*^+^) were included in four ontologies of the corpus (Table 3), which means that their expressivity corresponds to the DL language S (*ALCR*^+^) provided that complement is included. Also, six of the learning ontologies of the corpus include role hierarchy (*H*). Inverse properties (*I*) are used in nine ontologies. Number restriction (*N*) or qualified number restriction (*Q*) is included in four ontologies. Constructors that refer to nominals (*O*) were found in five ontologies. Finally, all the ontologies of the corpus include data types (*D*). These language features result in higher expressivity. In short, our analysis shows that the corpus includes ontologies with a range of expressivities. Also, it is worth studying the expressivity of the corpus in comparison to the OWL 2 standard profiles.

## OWL 2 constructors of the corpus and the OWL 2 RL profile

The OWL 2 RL profile includes a subset of OWL 2 constructors recommended by the W3C. In this section, we compare the constructors of the OWL 2 RL profile with those of the corpus. We check which OWL 2 RL constructors are used in the corpus and which ones are not used.

### OWL 2 RL constructors used in the corpus

The corpus includes OWL 2 RL constructors that belong to different categories. However, in some situations, the corpus includes “old” OWL constructors as well. These old OWL constructors are the predecessors of the OWL 2 RL constructors and each of these old OWL constructors and its corresponding OWL 2 constructor refer to the same DL constructor. Hence, in cases where an old OWL constructor was found, it was interpreted as an OWL 2 constructor.

#### Predefined class expressions

All predefined class expressions of OWL 2 RL, except the empty class owl:Nothing, were found in the corpus. The universal class (or top concept in DLs) owl:Thing is used as the superclass of all the other classes. The universal class owl:Thing includes all the individuals of an ontology. For example, in the *instOntology.owl* ontology, the class expression in (6) specifies that the class Research_Interest is a subclass of the universal class owl:Thing.

6 $$ {\texttt{SubClassOf}}\left( {{\texttt{:Research}}\_{\texttt{Interest \quad owl:Thing}}} \right) $$

Therefore, according to (6), instances (individuals) of the class Research_Interest become individuals of the universal class owl:Thing as well. The concepts in a domain are defined as named classes. In the above example, Research_Interest is a named class that is specific to the *instOntology.owl* learning ontology.

#### Boolean connectives and enumeration

The Boolean connectives intersection IntersectionOf() and enumeration OneOf() were found in both the OWL 2 RL profile and in the corpus. The connective IntersectionOf () has been used in the *university.owl* ontology. For example, the statement in (7) specifies that every artificial intelligence (AI) department is fully defined as a computer science (CS) department that has the research area AI.

7 $${\texttt{EquivalentClasses}}\left( {{\texttt{:AI}}\_{\texttt{Dept ObjectIntersectionOf}}\left( {{\texttt{ObjectHasValue}}\left( {{\texttt{:hasResearchArea :AI}}} \right){\mkern 1mu} {\texttt{ :CS}}\_{\texttt{Department}}} \right)} \right)$$

Enumeration OneOf () has only been used in the *ecs.owl* ontology of the corpus with a single value for each enumeration: OneOf (“A27″^^xsd:string), OneOf (“A41″^^xsd:string) and OneOf (“A47″^^xsd:string).

#### Object and data property restrictions

Both the OWL 2 RL profile and the corpus include data property restrictions. The OWL data property constructors domain() and range() used in the *university.owl* ontology correspond to the OWL 2 RL data property constructors DataPropertyDomain() and DataPropertyRange(), and they can be directly substituted by them in an OWL 2 ontology. For example, the domain and range restrictions of the data property hasTenure can be specified as shown in (8).

8 $$ \begin{aligned}&{\texttt{Declaration}}\left( {{\texttt{DataProperty}}\left( {:{\!\texttt{hasTenure}}} \right)} \right) \hfill \\ &{\texttt{DataPropertyDomain}}\left( {:{\!\texttt{hasTenure }}:{\!\texttt{TeachingFaculty}}} \right) \hfill \\& {\texttt{DataPropertyRange}}\left( {:{\!\texttt{hasTenure\ xsd}}:{\!\texttt{boolean}}} \right) \hfill \\ \end{aligned} $$

The data property constructors domain() and range() have also been used in the *uni_bench.owl* ontology to specify the domain and the range of the data property emailAddress.

All object property restrictions of the OWL 2 RL profile, except self-restriction ObjectHasSelf(), were found in the corpus. Universal quantification ObjectAllValuesFrom() was found in seven ontologies of the corpus and existential quantification ObjectSomeValuesFrom() in six ontologies of the corpus (Table 2).

Individual value restriction ObjectHasValue() has been used in a few ontologies of the corpus. This restriction appears in the *university.owl* ontology twice. It is used to specify that AI is a research area of the computer science department as shown in (9) below.

9 $$\begin{aligned} &{\texttt{ObjectPropertyAssertion}}\left( {:{\texttt{\!hasResearchArea }}:{\!\texttt{CS}}\_{\texttt{Department }}:{\!\texttt{AI}}} \right); \hfill \\ &{\texttt{ObjectHasValue}}\left( {:{\!\texttt{hasResearchArea }}:{\!\texttt{AI}}} \right) \hfill \\ \end{aligned}$$

Again, it has been used to specify that a teaching faculty has no tenure as shown in (10) below.

10 $$\begin{aligned} &{\texttt{ObjectPropertyAssertion}}\left( {:{\!\texttt{hasTenure }}:{\!\texttt{TeachingFaculty }}:{\!\texttt{False}}} \right); \hfill \\ &{\texttt{ObjectHasValue}}\left( {:{\!\texttt{hasTenure }}:{\!\texttt{False}}} \right) \hfill \\ \end{aligned}$$

The HERO Ontology also includes individual value restriction to specify three different situations. Firstly, it has been used to specify that a dean who is a technical staff has a doctorate degree (11).

11 $$\begin{aligned}& {\texttt{SubClassOf}}\left( {:{\!\texttt{Dean }}:{\!\texttt{TechnicalStaff}}} \right); \hfill \\ &\left.{\texttt{SubClassOf}}\left( {:{\!\texttt{Dean ObjectHasValue}}\left( {:{\!\texttt{HasDegree }}:{\!\texttt{doctorate}}} \right)} \right)\right) \hfill \\ \end{aligned}$$

Secondly, in the statement (12) below, it has been used to specify that a degree that is a deliverable is obtained by a doctorate.

12 $$\begin{aligned} &{\texttt{SubClassOf}}\left( {:{\!\texttt{Degree }}:{\!\texttt{Deliverable}}} \right) \hfill \\ &{\texttt{SubClassOf}}\left( {:{\!\texttt{Degree ObjectHasValue}}\left( {:{\!\texttt{ObtainedBy }}:{\!\texttt{doctorate}}} \right)} \right) \hfill \\ \end{aligned}$$

Finally, in the statement (13) below, it has also been used to specify that a registrar who is a department staff works with a chair.

13 $$\begin{aligned} &{\texttt{SubClassOf}}\left( {:{\!\texttt{Registrar }}:{\!\texttt{DepartmentStaff}}} \right) \hfill \\ &{\texttt{SubClassOf}}\left( {:{\!\texttt{Registrar ObjectHasValue}}\left( {:{\!\texttt{WorksWith }}:{\!\texttt{Chair}}} \right)} \right) \hfill \\ \end{aligned}$$

The individual value restriction ObjectHasValue() was also found in the *instOntology.owl* ontology. The statement (14) below specifies that a teacher who is a person has a PhD qualification.

14 $$\begin{aligned} &{\texttt{SubClassOf}}\left( {:{\!\texttt{Teacher }}:{\!\texttt{Person}}} \right) \hfill \\ &{\texttt{SubClassOf}}\left( {:{\!\texttt{Teacher ObjectHasValue}}\left( {:{\!\texttt{hasQualification }}:{\!\texttt{PhD}}} \right)} \right) \hfill \\ \end{aligned}$$

#### Object and data property expressions

OWL 2 properties are used to state property expressions (Motik et al. 2009). A named object property expression can be used to connect the individuals of a domain. For example, the statement (14) below from the *university.owl* ontology specifies that an AI student has a professor in HCI or AI as an advisor. The named object property expression hasAdvisor is used in (15) to specify that John has Peter as his advisor.

15 $$\begin{aligned} &{\texttt{Declaration}}\left( {{\texttt{ObjectProperty}}\left( {:{\!\texttt{hasAdvisor}}} \right)} \right) \hfill \\ &{\texttt{SubClassOf}}\left( {:{\!\texttt{AIStudent ObjectSomeValuesFrom}}\left( {:{\!\texttt{hasAdvisor }}:{\texttt{ProfessorInHCIorAI}}} \right)} \right) \hfill \\ &{\texttt{ObjectPropertyAssertion}}\left( {:{\!\texttt{hasAdvisor }}:{\!\texttt{John }}:{\!\texttt{Peter}}} \right) \end{aligned}$$

Similarly, a named data property expression can be used to connect an individual with a literal. For example, the statement (16) below from the *university.owl* ontology features a named data property expression hasTenure which is used to specify that Peter has tenure.

16 $${\texttt{DataPropertyAssertion}}\left( {{\texttt{hasTenure }}:{\!\texttt{Peter }}:{\!\texttt{True}}} \right)$$

#### Class expressions

A number of class expression constructors that are included in the OWL 2 RL profile were found in the corpus as well. As shown in (17) below, the class expression constructor SubClassOf() is used in the *university.owl* ontology to specify that computer science students are a subclass of students.

17 $${\texttt{SubClassOf}}\left( {:{\!\texttt{CS}}\_{\texttt{Student }}:{\!\texttt{Student}}} \right)$$

The equivalent class expression constructor EquivalentClasses() as shown in (18) has been used in the *university.owl* ontology to specify that the class AI_Dept is equivalent to the class CS_Department which has AI as a research area.

18 $${\texttt{EquivalentClasses}}\left( {:{\!\texttt{AI}}\_{\texttt{Dept ObjectIntersectionOf}}\left( {{\texttt{ObjectHasValue}}\left( {:{\!\texttt{hasResearchArea }}:{\!\texttt{AI}}} \right) \, :{\!\texttt{CS}}\_{\texttt{Department}}} \right)} \right)$$

In the *university.owl* ontology, it has also been stated that the class AssistantProfessor and the class Professor are disjoint as shown in (19).

19 $${\texttt{DisjointClasses}}\left( {:{\!\texttt{AssistantProfessor }}:{\!\texttt{Professor}}} \right)$$

#### Object properties

In order to specify object properties, OWL 2 RL includes the subobject property constructor SubObjectPropertyOf(), the object property domain constructor ObjectPropertyDomain() and the object property range constructor ObjectPropertyRange(). Similarly, the corpus includes the uses of the following OWL property constructors: subPropertyOf (), PropertyDomain() and PropertyRange() to specify object and data properties. For example, in the *uni_bench.owl* ontology, it is stated that the object property doctoralDegreeFrom is a subobject property of degreeFrom, as shown in (20).

20 $${\texttt{SubObjectPropertyOf}}\left( {:{\!\texttt{doctoralDegreeFrom }}:{\!\texttt{degreeFrom}}} \right)$$

Additional object property constructors were found in the corpus (Table 2). The OWL functional property constructor FunctionalProperty() that is similar to the OWL 2 RL functional object property constructor FunctionalObjectProperty() was found in both the *ecs.owl* ontology and the *HERO_ONTOLOGY_V 25.06.2013.owl* ontology. The statement in (21) specifies that each individual teacher is hired by at most one faculty.

21 $$\begin{aligned} {\texttt{FunctionalObjectProperty}}\left( {:{\!\texttt{IsHiredBy}}} \right)) \hfill \\ {\texttt{ObjectPropertyDomain}}\left( {:{\!\texttt{IsHiredBy }}:{\!\texttt{Teacher}}} \right) \hfill \\ {\texttt{ObjectPropertyRange}}\left( {:{\!\texttt{IsHiredBy }}:{\!\texttt{Faculty}}} \right) \hfill \\ \end{aligned}$$

The OWL inverse functional property constructor InverseFunctionalProperty() that is similar to the OWL 2 RL inverse object functional property constructor InverseFunctionalObjectProperty() was also found in the *HERO_ONTOLOGY_V 25.06.2013.owl* ontology. For example, (22) states that the inverse of the property Teaches is functional in this ontology.

22 $${\texttt{InverseFunctionalObjectProperty}}\left( {:{\!\texttt{Teaches}}} \right)$$

The OWL transitive property constructor was found in the *uni*-*bench.owl*. The transitive property constructor TransitiveProperty() is similar to the OWL 2 RL transitive object property constructor TransitiveObjectProperty(). Using the TransitiveObjectProperty() constructor, the property subOrganizationOf is defined to be transitive.

23 $${\texttt{TransitiveObjectProperty}}\left( {:{\!\texttt{subOrganizationOf}}} \right)$$

Similarly, the *university.owl* ontology also includes the TransitiveObjectProperty() constructor as shown in (24). It is used to specify that a university A is affiliated with another university C whenever A is affiliated with a university B and B is affiliated with C.

24 $${\texttt{TransitiveObjectProperty}}\left( {:{\!\texttt{affiliatedWith}}} \right)$$

#### Assertions

Assertions provide facts about individuals (Smith et al. 2004). Both the OWL 2 RL profile and the corpus include the uses of some constructors to make assertions about individuals or instances of OWL classes. The class assertion constructor ClassAssertion(C a) has been used in the corpus to specify the individuals of each class. For example, in the *MQ_Ontology.owl* ontology, the class assertion shown in (25) below states that ISYS114 is a unit.

25 $${\texttt{ClassAssertion}}\left( {:{\!\texttt{Unit :ISYS114}}} \right)$$

Object property assertions are made by using the OWL 2 constructor ObjectPropertyAssertion(PN a_1_ a_2_*)*. For example, in the *Macquarie.owl* ontology, the object property assertion shown in (26) states that the unit ISYS114 has particular lecture slides for week 1.

26 $${\texttt{ObjectPropertyAssertion}}\left( {:{\!\texttt{hasLectureSlides :ISYS114 :ISYS114LectureSlidesWk1}}} \right)$$

Again, the data properties are asserted using the constructor DataPropertyAssertion(R a v). For example, the following assertion (27) states that the unit ISYS114 is worth three credit points.

27 $$ {\texttt{DataPropertyAssertion(:creditPoints\,:ISYS114}}\ ^{\prime \prime } 3^{\prime \prime \wedge \wedge}{\texttt{xsd}}:\,{\texttt{integer)}} $$

### OWL 2 RL constructors not used in the corpus

Some of the constructors of OWL 2 RL were not found in the corpus. In the following, we discuss them in more detail and their potential use in learning ontologies.

#### Class expressions

The constructor for pairwise disjoint classes DisjointClasses() is included in the OWL 2 RL profile, but it was not found in the corpus. Also, even though many predefined class expressions of OWL 2 RL were found in the corpus, the empty class constructor (or the bottom concept in DLs) owl:Nothing was not found. The empty class constructor owl:Nothing is used to define terminal classes in a class hierarchy.

#### Boolean connectives and enumeration

The OWL 2 RL profile includes Boolean connectives for union: ObjectUnionOf() and DataUnionOf() and for complement: ObjectComplementOf() and DataComplementOf(). However, the corpus does not include these connectives or variants thereof: UnionOf() and ComplementOf(). In spite of that, the W3C has proposed three situations where union can be used (Motik et al. 2012).

1. Union of data ranges can be used to create a new data range by combining two or more data types. For example, xsd:string and xsd:integer can be joined as shown in (28) below to create a new data range with both xsd:string and xsd:integer.
  28 $$ {\texttt{DataUnionOf}}\left( {{\texttt{xsd}}:{\!\texttt{string\ xsd}}:{\!\texttt{integer}}} \right) $$
2. A union of class expressions can be used to form a new class that contains all the individuals that are instances of at least one of those class expressions (Motik et al. 2012). For example, the class expression in (29) could be used to create a new class that consists of all the individuals that are instances of either an assignment or a quiz.
  29 $${\texttt{ObjectUnionOf}}\left( {:{\!\texttt{Assignment }}:{\!\texttt{Quiz}}} \right)$$
3. A disjoint union of class expressions states that a class is the pairwise disjoint union of one or many class expressions (Motik et al. 2012). For example, the assertion shown in (3) below states that each assessment is either an assignment or an exam. Also, each assignment is an assessment, each exam is an assessment, and nothing can be both an assignment and an exam.
  30 $${\texttt{DisjointUnion}}\left( {:{\!\texttt{Assessment }}:{\!\texttt{Assignment }}:{\!\texttt{Exam}}} \right)$$

The W3C shows that complement can be used in two different ways: to specify the complement of class expressions using the constructor ObjectComplementOf() and to specify the complement of data ranges using the constructor DataComplementOf() (Motik et al. 2012). A complement of class expressions consists of all individuals that are not instances of that class expression. For example, the complement class expression in (31) specifies all those things that are not instances of the class Assignment.

31 $${\texttt{ObjectComplementOf}}\left( {:{\!\texttt{Assignment}}} \right)$$

A complement of a data range can be specified using the constructor DataComplementOf() and consists of all the tuples of literals that are not contained in the given data range (Motik et al. 2012). For example, in the statement in (32) describes literals that are not positive integers.

32 $${\texttt{DataComplementOf}}\left( {{\texttt{xsd}}\,:{\!\texttt{positiveInteger}}} \right)$$

#### Object and data property restrictions

The self-restriction ObjectHasSelf() is included in OWL 2 RL, but it was not found in the corpus. A self-restriction includes an object property expression (OPE). In addition, self-restriction includes all those individuals that are connected via an OPE to themselves (Motik et al. 2012). For example, the statements in (33) below specify that Mary loves herself.

33 $$\begin{aligned} &{\texttt{ObjectHasSelf}}\left( {:{\!\texttt{loves}}} \right) \hfill \\ &{\texttt{ObjectPropertyAssertion}}\left( {:{\!\texttt{loves }}:{\!\texttt{Mary }}:{\!\texttt{Mary}}} \right) \hfill \\ \end{aligned}$$

Even though OWL 2 RL includes all the OWL 2 RL data property restrictions, the corpus did not include any of them. OWL 2 RL includes data property restrictions that are similar to the object property restrictions except that there is no data property constructor for specifying reflexivity.

#### Object properties

The OWL 2 RL profile allows for property chain inclusion, however, we did not find any examples in the corpus. For instance, in the statement shown in (34), the property chain inclusion constructor ObjectPropertyChain(OPE_1 _… OPE_n_) can be used to specify that the extension of one object property expression is included in the extension of another property expression (Motik el al. 2012).

34 $${\texttt{SubPropertyOf}}\left( {{\texttt{ObjectPropertyChain}}\left( {:{\!\texttt{locatedIn }}:{\!\texttt{partOf}}} \right) \, :{\!\texttt{locatedIn}}} \right)$$

The equivalent object property constructor EquivalentObjectProperties(OPE_1_… OPE_n_) can be used to specify that all of the OPEs from 1 to n are semantically equivalent to each other (Motik et al. 2012). Therefore in specifying domain information in an ontology, one OPE can be replaced with another OPE. For example, in the learning domain, this constructor could be used to state that the properties hasTeacher and hasLecturer are equivalent properties as shown in (35).

35 $${\texttt{EquivalentObjectProperties}}\left( {:{\!\texttt{hasTeacher}}\,other Onto:{\!\texttt{hasLecturer}}} \right)$$

Pairwise disjoint properties are also included in the OWL RL profile; however, they were not found in the corpus. The disjoint object property constructor DisjointObjectProperties(OPE_1_… OPE_n_) can be used to specify that all of the OPEs from 1 to n are pairwise disjoint (Motik et al. 2012). For example, in the learning domain, we could specify that the object properties hasFinalExam and hasAssignment are disjoint as shown in (36).

36 $${\texttt{DisjointObjectProperties}}\left( {:{\!\texttt{hasFinalExam }}:{\!\texttt{hasAssignment}}} \right)$$

Reflexivity is an important property in general, however, the reflexivity object property constructor ReflexiveObjectProperty(OPE) of OWL 2 is not included in OWL 2 RL and was not found in the corpus. This constructor says that the OPE is reflexive. Hence, each individual that is connected by OPE refers to itself (Motik et al. 2012). Similarly, The irreflexivity object property constructor IrreflexiveObjectProperty(OPE) says that the OPE is irreflexive, that is, no individual is connected by the OPE to itself (Motik et al. 2012). For example, in the learning domain, we could specify that the object property prerequisiteOf is irreflexive as shown in (37).

37 $${\texttt{IrreflexiveObjectProperty}}\left( {:{\!\texttt{prerequisiteOf}}} \right)$$

The object property symmetry constructor SymmetricObjectProperty(OPE) states that the OPE is symmetric. That is, if x is connected to y by an OPE, then y is also connected to x by the same OPE. For example, in the learning domain, suppose that two particular subjects should be studied by a student in the same semester, then the OPE corequisiteOf could be used as shown in (38).

38 $${\texttt{SymmetricObjectProperty}}\left( {:{\!\texttt{corequisiteOf}}} \right)$$

The object property asymmetry constructor AsymmetricObjectProperty(OPE) states that the OPE is asymmetric. That is, if x is connected by an OPE to y, then y cannot be connected to x by the same OPE. For example, in the learning domain if one unit is the prerequisite of another unit, then, the second unit cannot be the prerequisite of the first unit as shown in (39).

39 $${\texttt{AsymmetricObjectProperty}}\left( {:{\!\texttt{prerequisiteOf}}} \right)$$

#### Assertions

The assertions used to compare individuals (equality or inequality) such as SameIndividual(a_1_… a_n_), DifferentIndividual(a_1 _a_2_) and DifferentIndividuals(a_1_… a_n_) were not found in the corpus. The corpus did not include negative property assertions of the form NegativeObjectPropertyAssertion(P a_1 _a_2_) and NegativeDataPropertyAssertion(R a v) either. The reason for this could be that the corpus includes ontologies that are specific to each institution. However, the above assertions would be more useful to compare elements of different ontologies. For example, the statement in (40) implies that John Miller and the lecturer of ISYS332 are the same individual in the two different ontologies Onto-A and Onto-B.

40 $$ {\texttt{SameIndividual}}\left( {Onto{\texttt{-}}A:{\!\texttt{JohnMiller}}\ Onto{\texttt{-}}B:{\!\texttt{ISYS332Lecturer}}} \right) $$

### OWL 2 RL vs the Learning Domain

Based on the above comparison between the constructors of the OWL 2 RL profile and the constructors used in the corpus, we observe that the corpus has fewer constructors than the OWL 2 RL profile. In particular, the OWL 2 RL profile includes all the different OWL 2 constructors that are associated with nominals (*O*). In addition to many object property restrictions, data property restrictions and assertions can be used in an ontology based on the OWL 2 RL profile. However, the corpus did not include those OWL 2 RL constructors that relate to nominals as well as object property restrictions, data property restrictions and assertions. Also, the corpus did not include object properties such as reflexivity, irreflexivity and role disjointness that contribute to the DL expressivity of *R*. Hence, we conclude that LD ontologies could be specified with a smaller set of OWL 2 constructors than those available in the OWL 2 RL profile. Such a subset of OWL 2 constructors may form an OWL 2 profile that is specific to the learning domain.

## OWL 2 learn profile

The new OWL 2 profile that we derived from the results of our analysis is called the OWL 2 Learn profile. The OWL 2 Learn profile includes the commonly used constructors of the corpus and some others that are infrequently used. We also include some OWL 2 constructors that are not used in the corpus. The inclusion or exclusion of a constructor depends on four factors: (1) the count (n) and the frequency (f) of the constructor, (2) the relative importance of the constructors, (3) the possibility of representing a constructor in an alternative way and, (4) the impact of the constructor on the computational complexity of the profile.

### The constructors included in the OWL 2 learn profile

The constructors that are commonly used in the corpus have higher scores of count (n) and frequencies (f). This shows that the commonly used constructors are required in many situations of the LD. Many infrequently used constructors are also included in the profile. The rationale for this is that some infrequently used constructors have a higher relative importance than some other infrequently used constructors. The constructor AllValuesFrom() is required to specify various basic situations of the domain. For example, in the *university.owl* ontology, the constructor AllValuesFrom() is used to specify that all the courses taken by students in the computer science department are computer science courses (see Table 2).

The inclusion of InverseObjectProperty() constructor provides the flexibility of navigating through an ontology in either direction of the OPE. For example, the OPE hasResource has the inverse property isResourceOf. Therefore, we can find the learning resources of a given unit using the OPE hasResource as well as the unit for which the learning resources are provided. The use of the inverse property makes query answering on a learning ontology easier.

Qualified cardinality restrictions are also infrequently used. They are more specific than non-qualified cardinality restrictions. Qualified cardinality restrictions clearly qualify what objects or data the restrictions are imposed on. Hence, specific results can be generated in query answering. For example, the qualified cardinality constructor MaxCardinality() of OWL has been used in the *university.owl* ontology. For example, the statement (41) specifies that a teaching faculty can take a maximum of three computer science courses. The OWL constructor MaxCardinality() correspond to the OWL 2 RL constructor ObjectMaxCardinality().

41 $${\texttt{MaxCardinality }}\left( { 3 \,{:}{\texttt{takesCourse }}\,{:}{\texttt{CS}}\_{\texttt{Course}}} \right)$$

The transitive object property constructor TransitiveObjectProperty() is infrequently used but it is included in the OWL 2 Learn profile. Transitivity cannot be naturally expressed using other constructors. For example, the OPE subOrganizationOf as shown in (42) of the *uni*-*bench.owl* ontology defines a transitive relationship between organisations. We can specify that every department is a suborganization of a faculty and every faculty is a suborganization of a university in the following way.

42 $$\begin{aligned} &{\texttt{TransitiveObjectProperty}}\left( {:{\!\texttt{subOrganizationOf}}} \right); \hfill \\ &{\texttt{ObjectPropertyDomain}}\left( {:{\!\texttt{subOrganizationOf }}:{\!\texttt{Organization}}} \right); \hfill \\ &{\texttt{ObjectPropertyRange}}\left( {:{\!\texttt{subOrganizationOf }}:{\!\texttt{Organization}}} \right); \hfill \\ &{\texttt{subOrganizationOf}}\left( {:{\!\texttt{Department }}:{\!\texttt{Faculty}}} \right);{\texttt{ subOrganizationOf}}\left( {:{\!\texttt{Faculty }}:{\!\texttt{University}}} \right) \end{aligned}$$

Another relevant example would be the prerequisite relationships between units of study where enrolment at certain units may require the completion of some other units. The transitive object property prerequisiteOf is used in the statements (43) below to specify that the unit COMP115 is a prerequisite of the unit COMP125 and the unit COMP125 is a prerequisite of the unit COM225.

43 $$\begin{aligned} {\texttt{TransitiveObjectProperty}}\left( {:{\!\texttt{prerequisiteOf}}} \right); \hfill \\ {\texttt{prerequisiteOf}}\left( {:{\!\texttt{COMP115 }}:{\!\texttt{COMP125}}} \right); \hfill \\ {\texttt{prerequisiteOf}}\left( {:{\!\texttt{COMP125 }}:{\!\texttt{COMP225}}} \right) \hfill \\ \end{aligned}$$

### Inclusion of unused constructors

As we discussed above, some OWL 2 constructors have not been used to express the domain knowledge in the corpus. However, some of them would seem to be required to specify specific domain knowledge. It could also be possible that some unused OWL 2 constructors may become useful to specify particular information of a future LD ontology. Therefore, we consider a few unused OWL/OWL2 constructors as candidates for inclusion in the OWL 2 Learn profile.

The OWL 2 constructor ObjectComplementOf() was not found in the corpus. However, the derived constructor SubClassOf() is found in the corpus and can be defined by means of disjunction and negation. That means, the OWL 2 constructor ObjectComplementOf() is indirectly an element of the OWL 2 Learn profile. Also, we include some additional datatypes in the OWL 2 Learn profile which were not found in the corpus (xsd:decimal, xsd:integer, xsd:long, xsd:int, xsd:float, xsd:double, xsd:string, xsd:Boolean, xsd:dateTime). Inclusion of these datatypes does not increase the expressivity of the profile, but they offer more syntactic freedom and flexibility for the ontology engineers.

### The excluded constructors

A number of OWL 2 constructors are excluded from the OWL 2 Learn profile. Many of them are not used in the corpus and some are infrequently used. Reflexive and irreflexive object properties, symmetric and asymmetric object properties are some of those excluded constructors. We think that those excluded constructors would rarely be required to specify an ontology in the LD.

Another main group of OWL 2 constructors that are excluded from the OWL 2 Learn profile are nominals. The OWL 2 constructors that refer to nominals are: ObjectOneOf(), DataOneOf(), ObjectHasValue() and DataHasValue(). They have been used only in three ontologies of the corpus. Even though the inclusion of nominals gives the ontology designers more syntactic freedom, it increases the computational complexity. While OWL 2 RL includes nominals, nominals have been excluded from several Semantic Web languages and are not used in some ontologies. For example, nominals have been excluded from the DL ontology proposed by Kepler et al. (2006). The DL reasoner *RacerPro* approximates nominals by atomic concepts (Haarslev et al. 2012).

With respect to the LD, we propose that domain information provided with the value constraint hasValue() can instead be specified using a primitive class. For example, in the *university.owl* ontology, the nominal AI could be replaced by a primitive class ResearchArea. Similarly, the *uni*-*bench.owl* and *lom.owl* ontologies also use nominals to specify domain information that can also be expressed using primitive classes.

### The expressivity of the OWL 2 learn profile

The expressivity of a DL language is determined by the DL constructors included in that language. Hence, to identify the expressivity of the OWL 2 Learn profile, we list each OWL 2 constructor or constructor group together with the corresponding DL constructor (Table 4). Accordingly, the OWL 2 Learn profile consists of all the included OWL 2 constructors and is more expressive that the DL language *ALC.* The profile includes transitive properties (*R* +) which lift the expressivity to the DL language *S (ALCR* +*)*, plus several other DL constructors that further increase the expressivity of the profile to *SHIQU(D)*. Since the symbol *C* for complement can be used in place of the symbols *UE* for union and existential quantification (Baader et al. 2003), the DL expressivity of OWL 2 Learn becomes *SHIQ(D)*.

**Table 4:** The constructors of the OWL 2 learn profile

**#**	OWL 2 constructor/s	DL constructor/s	DL language		
1	Thing, Nothing	Top—$\top$, Bottom—$\perp$	*FL* ^−^	*AL* (with atomic negation), *ALC* (with full negation)	*S/(ALCR* +*)*
2	Class	Atomic concept—A			
3	ObjectIntersectionOf	Conjunction—⊓			
4	ObjectAllValuesFrom	Universal restriction—∀			
5	ObjectSomeValuesFrom	Limited/Full Existential restriction—∃			
6	ObjectProperty	Atomic role—R			
7	ClassAssertion, ObjectPropertyAssertion	Assertions C(a), R(b, c)			
8	ObjectComplementOf	Negation—¬			
9	TransitiveObjectProperty	Transitive role—Tr (R)	*R*+		
10	SubObjectPropertyOf SubDataPropertyOf	Role hierarchy—H	*H*		
11	InverseObjectProperties	Inverse role—I	*I*		
12	Max/Min/Exact Cardinality	Qualified cardinality restrictions—Q	*Q*		
13	DisjointClasses	Disjunction—⊔	*U*		
14	DataProperty, DataPropertyAssertion, xsd:{integer, string, …}	Data {types, values} (D)	*D*		
Expressivity of the OWL 2 Learn Profile			*SHIQ(D)*		

We note that the OWL 2 Learn profile has a lower expressivity than OWL 2 RL. Still, it can be used to specify all the learning ontologies of the corpus. The three ontologies: *university.owl, uni*-*bench.owl* and *lom.owl* that include nominals can also be specified in OWL 2 Learn with minor changes by converting the nominals to instances of primitive concepts.

### The usage of the OWL 2 learn profile

To demonstrate the usage of OWL 2 Learn profile, an excerpt of the Macquarie University (MQ) ontology is given in Table 5. The MQ ontology is compliant with the OWL 2 Learn profile. The excerpt includes different statements from the MQ ontology that use different OWL 2 Learn constructors from Table 4. Firstly, the example includes the declaration of a class Person and a class AssessmentTask using the OWL 2 Learn constructor Class() as shown in (44).

44 $${\texttt{Declaration}}\left( {{\texttt{Class}}\left( {:{\!\texttt{Person}}} \right)} \right) \,$$

**Table 5:** An Excerpt of the MQ Ontology

An excerpt of the MQ ontology	The OWL 2 learn constructor	Ref to the Table 4
Declaration(Class(:Person))	Class	#2
EquivalentClasses(:Person ObjectSomeValuesFrom(:isAssociatedTo :Organisation))	ObjectSomeValuesFrom	#5
SubClassOf(:Person owl:Thing)	Owl:Thing SubClassOf	#1
SubClassOf(:Staff :Person)		
SubClassOf(:Student :Person)		
SubClassOf(:TeachingStaff :Staff)		
Declaration(Class(:Topic))		
SubClassOf(:Topic owl:Thing)		
SubClassOf(:Topic ObjectAllValuesFrom(:hasScheduled :TopicList))	ObjectAllValuesFrom	#4
Declaration(Class:TopicList))		
SubClassOf(:TopicList owl:Thing)		
SubClassOf(:TopicList ObjectSomeValuesFrom(:hasScheduledTopic :Topic))	ObjectSomeValuesFrom	#5
SubClassOf(:Unit owl:Thing)		
Declaration(Class(:AssessmentTask))		
EquivalentClasses(:AssessmentTask ObjectIntersectionOf(owl:Thing	EquivalentClasses	
ObjectSomeValuesFrom(:isWrittenBy :TeachingStaff)	ObjectIntersectionOf	#3
ObjectExactCardinality(1 :isAssessmentMethodOf :Unit)))	ObjectSomeValuesFrom	#5
	QualifiedExactCardinality	#12
DisjointUnionOf(:AssessmentTask :Assignment :Tutorial :FinalExam))	DisjointUnionOf	#13
	DisjointClasses	
DisjointClasses( :Assignment :FinalExam)		
SubClassOf(:AssessmentTask owl:Thing)		
SubClassOf(:FinalExam :AssessmentTask)		
SubClassOf(:Assignment :AssessmentTask)		
Declaration(ObjectProperty(:hasPrerequisite))	ObjectProperty	#6
TransitiveObjectProperty(:hasPrerequisite)	TransitiveObjectProperty	#9
InverseObjectProperties(:isPrerequisiteOf :hasPrerequisite)	InverseObjectProperties	#11
ObjectPropertyDomain(:hasPrerequisite :Unit)	ObjectPropertyRange	
ObjectPropertyRange(:hasPrerequisite :Unit)	ObjectPropertyDomain	
Declaration(ObjectProperty (:commitsTo))		
InverseObjectProperties(:commitsTo :isCommitedBy)		
ObjectPropertyDomain(:commitsTo :Student)		
ObjectPropertyRange(:commitsTo :Enrolment)		
ObjectPropertyRange(:commitsTo ObjectMaxCardinality(5 :commitsTo :Enrolment))	MaxQualifiedCardinality	#12
Declaration(ObjectProperty:assignmentOf))		
SubObjectPropertyOf(:assignmentOf :isAssessmentMethodOf)	SubObjectPropertyOf	#10
InverseObjectProperties(:has Assignment:assignmentOf)		
Declaration(DataProperty(:assignmentMark))	DataProperty	#14
SubDataPropertyOf(:assignmentMark :assessmentMark)	SubDataPropertyOf	#10
DataPropertyDomain(:assignmentMark :AssignmentSubmission)		
DataPropertyRange( :assignmentMark^^ xsd:integer)		
ClassAssertion(:Unit :ISYS114)	ClassAssertion	#7
ObjectPropertyAssertion(:isTutorOf :JohnParker :ISYS114)	ObjectPropertyAssertion	#7

Moreover, it includes the subclasses of the class Person and AssessmentTask. Secondly, it includes the declaration of some object properties such as hasPrerequsite as shown in (45) followed by other type of properties.

45 $${\texttt{Declaration}}\left( {{\texttt{ObjectProperty}}\left( {:{\!\texttt{hasPrerequisite}}} \right)} \right)$$

Thirdly, the data property assignmentMark is declared as a subproperty of the property assessmentMark. Finally, a class assertion and an object property assertion is given.

## Conclusion

Our analysis shows that a corpus of 14 learning ontologies includes a subset of OWL 2 constructors. This subset is different from the OWL 2 constructors in the OWL 2 RL profile. Predominantly, the OWL 2 RL profile includes all the nominal constructors whereas the corpus includes only a few occurrences of nominals. Those occurrences of nominal could also be represented using primitive classes. Since the OWL 2 constructors of the corpus form a subset of the OWL 2 RL profile, we consider this subset as a new profile and call it OWL 2 Learn profile. The OWL 2 Learn profile includes the great majority of the OWL 2 constructors that are used in the corpus and is sufficient to build all the ontologies in it with small modifications.

Our analysis includes a comparison of the OWL/OWL 2 constructors in the corpus with those of the OWL 2 RL profile. This comparison gives the ontology designers an insight into the use of OWL 2 constructors in existing LD ontologies. The new OWL 2 Learn profile has the expressivity of *SHIQ(D)* that is lower than the expressivity of the OWL 2 RL profile *(SROIQ(D))*. Hence, the potential ontology designers may select a DL-based reasoner that supports OWL 2 RL, for reasoning and querying an OWL 2 Learn ontology. KAON2 (Hufstadt et al. 2004) would be an ideal candidate for a reasoning engine for the OWL 2 Learn profile as they have the same expressivity. We are currently compiling a set of benchmark queries that comply with the OWL 2 Learn profile. We also plan to develop a suitable query language for the OWL 2 Learn profile and to further investigate its applications in learning management systems.

## Abbreviations

AL: attributive language
ALC: attributive language with complement
DL: description logic
DPE: data Property expression
XML: extensible mark-up language (XML)
FL: frame language
HERO: higher Education Reference Ontology
KR: knowledge representation
LD: learning domain
OWL: web ontology language
OPE: object property expression
RDF: resource description framework
UML: unified modelling language
W3C: World Wide Web Consortium

## Footnotes

1 http://rpc295.cs.man.ac.uk:8080/repository/browser.
